# Housing quality and behavior affect brain health and anxiety in healthy Japanese adults

**DOI:** 10.1038/s41598-021-91363-4

**Published:** 2021-06-07

**Authors:** Juan Cesar D. Pineda, Keisuke Kokubun, Toshiharu Ikaga, Yoshinori Yamakawa

**Affiliations:** 1 Open Innovation Institute, Kyoto University, Kyoto, Japan; 2 Faculty of Science and Technology, Keio University, Yokohama, Kanagawa Japan; 3 ImPACT Program of Council for Science, Technology and Innovation (Cabinet Office, Government of Japan), Chiyoda, Tokyo, Japan; 4 Institute of Innovative Research, Tokyo Institute of Technology, Meguro, Tokyo, Japan; 5 Office for Academic and Industrial Innovation, Kobe University, Kobe, Japan; 6 Brain Impact General Incorporated Association, Kyoto, Japan

**Keywords:** Diagnostic markers, Predictive markers, Development of the nervous system, Stress and resilience, Synaptic plasticity, Human behaviour

## Abstract

Countless studies in animals have shown how housing environments and behaviors can significantly affect anxiety and brain health, giving valuable insight as to whether this is applicable in the human context. The relationship between housing, behavior, brain health, and mental wellbeing in humans remains poorly understood. We therefore explored the interaction of housing quality, weekend/holiday sedentary behavior, brain structure, and anxiety in healthy Japanese adults. Whole-brain structural magnetic resonance imaging (MRI) methods based on gray matter volume and fractional anisotropy were used as markers for brain health. Correlation tests were conducted, and then adjusted for multiple comparisons using the False Discovery Rate method. Housing quality and weekend/holiday sedentary behavior were associated with fractional anisotropy, but not with gray matter volume. Fractional anisotropy showed significant associations with anxiety. Lastly, both weekend/holiday sedentary behavior and housing quality were indirectly associated with anxiety through fractional anisotropy. These results add to the limited evidence surrounding the relationship among housing, behavior, and the brain. Furthermore, these results show that behavior and housing qualities can have an indirect impact on anxiety through neurobiological markers such as fractional anisotropy.

## Introduction

The ill effects of the coronavirus disease 2019 (COVID 19) have spilled over into various mental health problems^1,2^, which may have been caused by strict lockdown and social distancing measures^3^. In response to the advent of new lifestyles, staying physically and mentally fit whilst staying at home have become of great interest. Living environments evoke various experiences and behavioral activity, which overtime considerably affect psychological dispositions^4^. This is highly evident in Enriched Environment (EE) experiments in animals. EEs are housing environments designed to increase physical, social, and cognitive activity. A substantial amount of literature has shown that exposure to EEs reduces anxiety^5–7^ and stress^8^. Such environments are also shown to have profound effects on brain plasticity, a key component in healthy brain aging. Histological analyses of animal brains post experiment showed an increase in neurons^9,10^, synaptic activity^11,12^, and overall brain volume^13,14^. These findings provide valuable insight as to whether EEs in the human context affect anxiety and brain health.

Multiple systematic reviews have shown the positive effects of physical activity on human structural neuroplasticity. In general, higher cardiorespiratory fitness or doses of physical activity seem to have protective effects on gray matter^15,16^ and white matter volumes^17–19^. Intriguingly, in light of the recent shift to home-based lifestyles, sedentariness is increasingly being shown as a separate risk factor. Multiple studies have shown that sedentary behavior is associated with brain atrophy, independent of physical activity^20–22^. Although the detrimental health effects of sedentariness can be reduced through physical activity, but this approach may be impractical for the general population. A study showed thatan individual needs to perform around 60-75 minutes of moderately intense physical activity throughout the day in order to reduce mortality risks associated to sedentary lifestyles, which is well beyond the guidelines set by the World Health Organization (150 minutes of moderately intense physical activity throughout the week)^23^. In terms of psychological outcomes, it was only until recently that researchers started to investigate the effects of sedentary behavior on anxiety. A prospective study showed that sitting for more than 42 hours per week is associated with a 31% increased risk of developing a mental disorder compared with sitting for less than 10.5 hours per week^24^. In a recent systematic review, sedentary behavior was shown to have positive associations with various mental health disorders, such as an increased risk for developing anxiety^25^.

In addition to physical activity and sedentariness, the efficacy of an EE also rests on its ability to stimulate extended social and cognitive behaviors^26^. Housing environments, however, comprise of several other “trivial” elements such as indoor thermal conditions, lighting, and humidity. These housing qualities are oftentimes overlooked but are shown to influence experimental outcomes^27^. For example, the standard housing temperature of 22 °C, compared to 30 °C, already puts mice under a certain degree of stress at baseline^28,29^.

In humans, the quality of housing varies across different socioeconomic stratums (SES). Census data from the American Housing Survey dated 1989–2001 shows that individuals who lived in substandard housing conditions belonged to lower income brackets^30^. In another longitudinal study (1995–2013), lead exposure was shown to be more concentrated in lower SES individuals and neighborhoods^31^.

Relatedly, SES is consistently shown to affect brain health^32^. Cross-sectional MRI analyses have shown that higher years of education and household income have positive associations with gray matter volume^33^ and white matter integrity^34,35^ in both children and adults. In a prospective study, a significant association was found between childhood SES and white matter integrity, measured during adulthood^36^. Given that SES affects brain structure across multiple age groups, it is possible that markers related to SES, such as housing qualities, may also have associations with brain structures.

Evidence on the effects of human housing qualities, such as indoor thermal conditions, on structural brain markers appears to be scant. A previous study by Dufford and Kim (2017), although not directly related to indoor thermal conditions, is the only study that investigated the impact of poor housing qualities on fractional anisotropy. Their study assessed housing quality in terms of structural defects, maintenance, childhood resources, safety hazards, and cleanliness. They found that individuals exposed to poor housing qualities had lower fractional anisotropy scores^37^.

The improvements in certain housing qualities, such as the installation of central heating, has been shown to reduce symptoms of anxiety^38,39^. In a randomized study, retrofit insulation improved mental health and was associated with fewer hospital visits^40^. In another study, air-conditioning had significant associations with anxiety^41^. In terms of brain function, an interventional study showed that the manipulation of ambient temperatures during fMRI scans activates brain regions related to behavioral adaptations to external stimuli^42,43^. Similarly, thermal stimulations were shown to activate specific brain regions related to positive and negative emotion^44^. In a randomized trial, hyperthermia affected executive function and reduced neural efficiency in healthy participants^45^. More notably, a study by Kim et al. investigated the effects of different thermal sensations on emotional responses by measuring brain activity. Kim and colleagues were able to show that different emotional responses can be attained by changing the ambient temperature. Pleasant emotions were seen when the ambient temperature was slightly warm, and unpleasant in all other cases^46^. The results from these studies already show, to a certain extent, that indoor environments affect the brain at the state level. The current evidence can be further enriched by investigating the effects on structural brain markers, as these markers are usually not affected by transient psychological conditions^47^.

**Figure 1:**
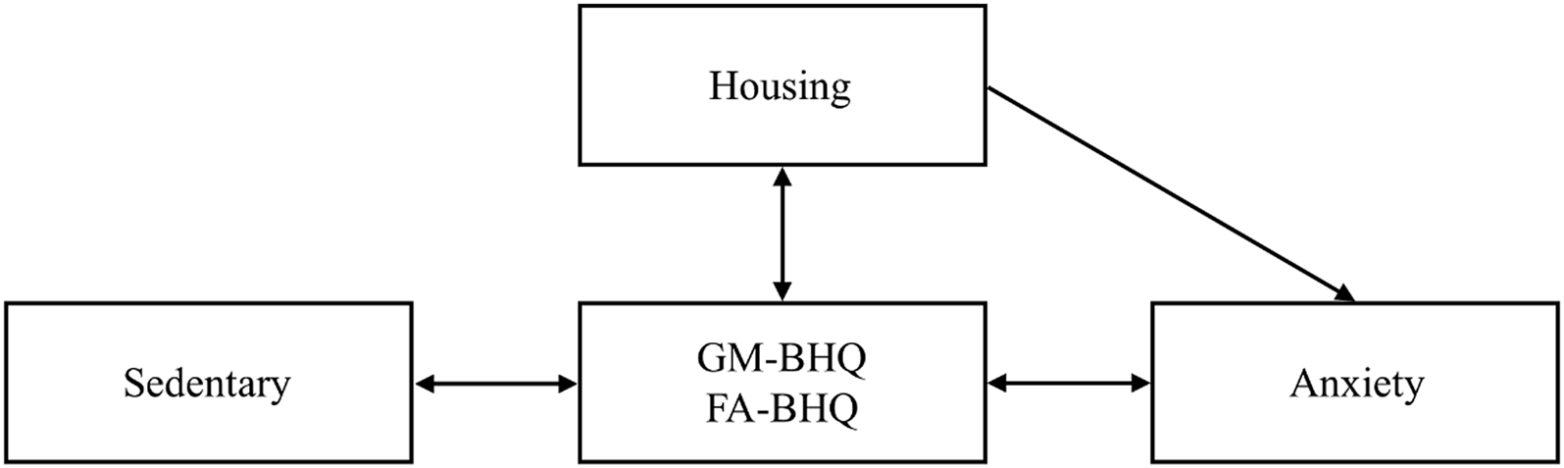
Hypothesized associations among housing quality, weekend/holiday sedentary behavior, brain health, and anxiety. The relationships shown below are assumptions based on the current literature, and are not in any way definitive or causal.

We also speculate that differences in behavioral choices during weekdays and weekend-days may have differential effects on anxiety, as one study showed that anxiety is more pronounced during the weekend^48^. Therefore, this study aimed to investigate the impact of housing quality and weekend/holiday sedentary behavior on the central nervous system, specifically on brain structure and anxiety (see Fig. 1).

## Materials and methods

### Subjects

One hundred and nine healthy individuals (47 females and 62 males), aged 25–69 [mean (M) ± standard deviation (SD): 48.239 ± 8.773 years] participated in the experiment, which was conducted in Kyoto University (Kyoto City, Japan) and the University of Tokyo (Tokyo City, Japan). The experiment required each participant to undergo an MRI brain scan and answer a set of questionnaires to assess their housing quality, their weekend/holiday sedentary behavior at home, and their current anxiety level.

This study was approved by the ethics committees of Kyoto University (approval number 27-P-13) and the University of Tokyo (Tokyo, Japan; approval number 402-2), and performed in accordance with the guidelines and regulations of the institute, respecting the Declaration of Helsinki. All participants gave written informed consent prior to participation, and participant anonymity was preserved.

### Housing quality and weekend/holiday sedentary behavior

This study focused on indoor insulation and space heating/cooling, which was measured using a housing quality assessment tool called the Comprehensive Assessment System for Built Environment Efficiency (CASBEE)^49–51^. This tool evaluates various housing qualities such as quietness, dampness or presence of mold, safety, peace of mind, indoor temperature, etc. Since this study focuses on indoor thermal environment, the following questions were included in the analysis: (1) “During summer, do you feel hot without using space cooling?” (2) “During winter, do you feel cold without using space heating?” Participants were asked to rate how hot/cold they felt during the summer/winter without using space cooling/heating facilities, using a four-point Likert scale (0—Extremely uncomfortable to 3—Very Comfortable). Higher scores indicate lower levels of discomfort.

Weekend/holiday sedentary behavior was measured using the International Physical Activity Questionnaire (IPAQ)^52^. Participants indicated the amount of time they spend sitting/lying down during weekend/holidays (in minutes).

### Anxiety

Anxiety was measured using the Brief Job Stress Questionnaire (BJSQ). Developed by the Japanese Ministry of Labor and health, this instrument has been widely used in research and practice in the field of workplace mental health in Japan^53^. The BJSQ asks the participant how he/she has felt during the past month, in terms of Liveliness, Irritability, Tiredness, Anxiety, Depression, and Physical complaints. The questions were answered through a three-point Likert scale (0—Strongly Agree to 3—Strongly Disagree). Three questions were related to Anxiety: “I have been full of energy”, “I have felt worried or insecure”, and “I have felt restless”. The sum of the scores of these 3 questions was used for analysis.

### MRI data acquisition

Similar to the previous MRI experiment by Nemoto and colleagues^54^, all magnetic resonance imaging (MRI) data were collected using a 3-T Siemens scanner (Verio, Siemens Medical Solutions, Erlangen, Germany or MAGNETOM Prisma, Siemens, Munich, Germany) equipped with a 32- or 64-channel head array coil at Kyoto University and the University of Tokyo. A high-resolution structural image was acquired using a three-dimensional (3D) T1-weighted magnetization-prepared rapid-acquisition gradient echo (MP-RAGE) pulse sequence. The parameters were as follows: repetition time (TR), 1900 ms; echo time (TE), 2.52 ms; inversion time (TI), 900 ms; flip angle, 9°; matrix size, 256 × 256; field of view (FOV), 256 mm; and slice thickness, 1 mm^54^.

DTI data were collected with spin-echo echo-planar imaging (SE-EPI) with GRAPPA (generalized autocalibrating partially parallel acquisitions). The image slices were parallel to the orbitomeatal (OM) line. The parameters were as follows: TR, 14,100 ms; TE, 81 ms, flip angle, 90°; matrix size, 114 × 114; FOV, 224 mm; slice thickness, 2 mm. A baseline image (b = 0 s/mm^2^) and 30 different diffusion orientations were acquired with a b value of 1000 s/mm^2^, similar to the study by Nemoto et al.^54^.

### MRI data analysis

The calculation of the gray matter volume in this study is identical to the calculation methods used in our previous neuroimaging study^54^. In summary, gray matter images were segmented from T1-weighted images using Statistical Parametric Mapping 12 (SPM12; Wellcome Trust Centre for Neuroimaging, London, UK) running on MATLAB R2015b (Mathworks Inc., Sherborn, MA, USA), followed by spatial normalization using diffeomorphic anatomical registration through an exponentiated lie algebra (DARTEL) algorithm^55^ and modulation to preserve the GM volume. All normalized, segmented, and modulated images were smoothed with an 8-mm full width at half-maximum (FWHM) Gaussian kernel. Additionally, intracranial volume (ICV) was calculated by summing the GM, white matter, and cerebrospinal fluid images for each subject. Proportional GM images were generated by dividing smoothed GM images by ICV to control for differences in whole-brain volume across participants. Using these proportional GM images, images for the mean and standard deviation (SD) across participants were generated. Then, we calculated the gray matter volume using the following formula: 100 + 15 × (individual proportional GM − mean)/SD. Regional GM quotients were then extracted using an automated anatomical labeling (AAL) atlas^56^ and averaged across regions to produce participant-specific gray matter volumes.

DTI data were preprocessed using FMRIB Software Library (FSL) 5.0.9^57^. First, all diffusion images were aligned with the initial b0 image, and motion correction and eddy current distortion correction was performed using eddy_correct. Following these corrections, FA images were calculated using dtifit. FA images were then spatially normalized into the standard Montreal Neurological Institute (MNI) space using FLIRT and FNIRT. FLIRT, a linear registration tool, was used to roughly align a set of brains to MNI space. Then FNIRT, a non-linear registration tool, was used to achieve better registration. After spatial normalization we smoothed the data with an 8-mm FWHM. Mean and SD images were generated from all the FA images, and both individual FA quotient images and GM-BHQ images were calculated. Individual FA quotient images were calculated using the following formula: 100 + 15 × (individual FA − mean)/SD. Regional FA quotients were extracted using Johns Hopkins University (JHU) DTI-based white-matter atlases and averaged across regions to produce participant-specific FA-BHQs^53^.

### Statistical analysis

Preliminary statistical analysis was performed using SPSS version 26 (IBM Corporation, Armonk, NY, USA) and AMOS. The preliminary analysis included four tests: (1) two Student’s t-test (two-tailed) to determine if the mean values of the predictor variables were strongly influenced by sex and the experiment location (Kyoto City or Tokyo City), (2) a chi-square test to determine if the experiment location and sex had significant distributional differences, (3) zero-order correlation analysis to find out if the predictor variables had associations with anxiety, and (4) partial correlation analysis to determine if the combination of housing and weekend/holiday sedentary behavior as a whole affected anxiety. The main analysis was a path analysis to explore the direct and indirect relationships among housing quality, weekend/holiday sedentary behavior, overall white matter integrity, overall gray matter volume, and anxiety. The path analysis was calculated using AMOS Version 26 (IBM Corp., Armonk, NY, USA). For brevity purposes, “Housing quality” was renamed to “Housing” and “Weekend/holiday sedentary behavior” was renamed to “Sedentary”.

### Ethics statement

The studies involving human participants were reviewed and approved by the Ethics Committees of Kyoto University (approval number 27-P-13) and the University of Tokyo (approval number 402). The patients/participants provided their written informed consent to participate in this study.

## Results

Results from the first t-test in Table 1 showed statistical mean difference between men and women when it comes to FA-BHQ (t = − 2.487, p = 0.014) and GM-BHQ scores (t = − 5.807, p < 0.001). The results from the chi-square test, also in Table 1, showed a p-value less than the 0.001 significance level, suggesting that there is a relationship between sex and experiment location.

**Table 1:** Student’s t test (two-tailed) and chi square test for the statistical difference between male and female participants.

	Male		Female		t	p
	Mean	SD	Mean	SD		
Age	49.610	8.912	46.430	8.335	1.901	0.060
FA-BHQ	99.180	4.291	101.090	3.488	− 2.487	0.014*
GM-BHQ	96.540	7.124	104.640	7.329	− 5.807	0.000***
Housing	3.190	1.587	3.490	1.502	− 0.986	0.326
Sedentary	366.450	220.707	379.790	227.037	− 0.309	0.758
Anxiety	4.650	1.700	4.020	2.142	1.696	0.093

	n	%	n	%	χ^2^	p
Kyoto	22	20%	36	33%	18.149	0.000***
Tokyo	40	37%	11	10%		

Results from the second t-test in Table 2 also showed significant mean differences between all continuous predictor variables and the location of the MRI system used for the experiment (except for Sedentary). Therefore, the succeeding correlation and path analyses controlled for age, sex, and experiment location.

**Table 2:** Student’s t test for the statistical difference between two experiment locations (Kyoto City, Tokyo City).

	Kyoto		Tokyo		t	p
	Mean	SD	Mean	SD		
Age	44.520	8.168	52.470	7.474	− 5.277	0.000***
FA-BHQ	101.710	3.356	98.060	3.942	5.219	0.000***
GM-BHQ	103.497	8.044	96.094	6.549	5.223	0.000***
Housing	3.620	1.497	2.980	1.556	2.188	0.031**
Sedentary	392.760	238.525	348.820	202.58	1.029	0.306
Anxiety	3.970	1.901	4.840	1.848	− 2.437	0.016*

Table 3 shows the descriptive statistics and results from the full and partial correlation tests. Subjects comprised a total of one hundred and nine participants (n = 109, 47 females and 62 males), aged 25–69 [mean (M) ± standard deviation (SD): 48.239 ± 8.773 years]. Due to the multiple comparisons (5 variables), p-values were corrected through FDR post-test. The adjusted p-value (q) was calculated using the standard threshold of 0.05. The following full correlations survived the FDR correction: Anxiety and Sedentary (r = 0.211, p = 0.028, q = 0.030), Housing and FA-BHQ (r = 0.237, p = 0.013, q = 0.035), Anxiety and Housing (r = − 0.243, p = 0.011, q = 0.040), and Anxiety and FA-BHQ (r = − 0.281, p = 0.003, q = 0.045).

**Table 3:** Descriptive statistics and correlations. n = 109. Correlations appear below the diagonal and partial correlations (controlled for age and sex, and experiment location) above diagonal. R values with an asterisk (*) denote significance after FDR correction.

Variable	Mean	SD	Anxiety	Housing	GM-BHQ	FA-BHQ	Sedentary
Anxiety	4.376	1.919	1.000	− 0.209*	0.013	− 0.206*	0.242
Housing	3.321	1.551	− 0.243	1.000	0.070	0.223*	− 0.063
GM-BHQ	100.033	8.233	− 0.134	0.081	1.000	0.153	− 0.05
FA-BHQ	100.000	4.060	− 0.281	0.237	0.483*	1.000	− 0.253*
Sedentary	372.202	222.512	0.211	− 0.044	0.026	− 0.153	1.000

Partial correlation coefficients are placed above the diagonal. After FDR correction, Anxiety was still associated with FA-BHQ (r = − 0.206, p = 0.034, q = 0.030), although marginal. Housing (r = 0.209, p = 0.031, q = 0.035), and Sedentary (r = 0.242, p = 0.013, q = 0.045) still remained significantly associated with Anxiety. There was a moderate, negative association between FA-BHQ (100.000 ± 4.060) and Sedentary (372.202 ± 222.512), which was statistically significant (r = − 0.253, p = 0.009, q = 0.050). This association was not seen in the prior zero-order correlation test (r = − 0.153, p = 0.112), indicating that age, sex, and experiment location played a significant role in controlling for the relationship between FA-BHQ and Sedentary. GM-BHQ did not have a significant association with anxiety in the full and partial correlation tests. Therefore, GM-BHQ was not included in the succeeding path analysis.

**Figure 2:**
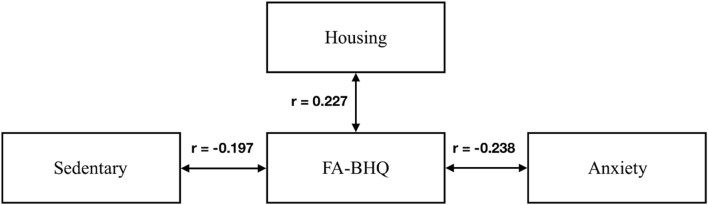
Path diagram for the resulting interactions among Sedentary, Housing, FA-BHQ, and anxiety, after controlling for Age and experiment location. FDR correction was applied using a threshold of 0.05. All pathways shown are significant.

To explore the interactions among housing, weekend/holiday sedentary behavior, brain health, and anxiety, we conducted a path analysis, as shown in Fig. 2. The figures of the standardized path coefficient, calculated using AMOS Version 26 (IBM Corp., Armonk, NY, USA), are also shown. The figures are the standardized path coefficient. FDR correction was also applied post-test. The adjusted p-value (q) was calculated using the standard threshold of 0.05. This test controlled for age and experiment location (sex was not used in the path model as it has a strong correlation with experiment location as shown in Table 1). All paths still showed significant associations after FDR correction.

The path analysis showed that Housing had an indirect association with Anxiety through FA-BHQ. Sedentary also had an indirect association with Anxiety through FA-BHQ. Sedentary did not have direct associations with Anxiety. Sedentary was marginally associated with FA-BHQ (r = − 0.197, p = 0.013, q = 0.01). Housing had significant associations with FA-BHQ (r = 0.227, p = 0.006, q = 0.020). FA-BHQ showed a negative direct association with Anxiety (r = − 0.238, p = 0.010, q = 0.015). Between Sedentary and Housing, Housing showed a stronger association with FA-BHQ.

## Discussion

Countless studies in animals have shown that exposure to EEs improves brain health through structural brain plasticity^5,6,9,10^ and promotes anxiolytic behaviors^5–7^. The convincing evidence begs the question as to whether the same applies in the human context. Human housing qualities are markedly influenced by SES, and recent evidence shows that SES affects brain structures across multiple age groups. It is therefore possible that markers related to SES may also affect brain structures. This exploratory study evaluated the effects of human housing quality and weekend sedentary behaviors on whole brain structure and anxiety. Whole brain structure was examined using two MRI measures based on gray matter volume and fractional anisotropy (called the GM-BHQ and FA-BHQ, respectively).

Our results reveal that housing quality and behavior are associated with white matter integrity, but not with whole brain gray matter volume. The null finding on gray matter volume seems to be in congruence with the previous study by Arnardottir et al.^21^. In their 5-year longitudinal cohort trial, only the decrease in white matter volume was associated with sedentary behavior. This shows a possibility that structural changes in white matter integrity in healthy adults is a sensitive biomarker that precedes gray matter volume loss. Interestingly, previous temporal studies have also posited that changes in white matter precedes volumetric changes in gray matter during normal aging^58,59^. Future neuroimaging studies may further investigate this speculation in order to shed light on the extent to which the changes in fractional anisotropy and gray matter volume are related.

Indices of white matter, such as fractional anisotropy, are said to be representations of brain integrity/resiliency^60,61^. Indeed, numerous observational and intervention studies have shown fractional anisotropy as a possible biomarker for psychological disorders such as anxiety. Kim et al.^62^ and Lu et al.^63^ demonstrated that trait anxiety in healthy individuals is inversely correlated with FA scores^62,63^. Tromp et al. showed that FA scores in Generalized Anxiety Disorder (GAD) patients was significantly lower compared to healthy controls^64^. Our findings, although cross-sectional, add to the extant literature on the relationship between white matter integrity and symptoms of anxiety in healthy individuals. This kind of study is particularly important, as the recent pandemic has greatly reduced and disrupted social connections and relationships, which has led to the surge in mental health issues^1-3^. It is possible that the effects could be more pronounced on vulnerable groups, such as communities or individuals on the lower end of the SES spectrum.

This study also presents a significant contribution to the identification of environmental risk factors of anxiety through neurobiological markers. More specifically, this study showed housing quality measures as possible surrogate markers for SES characteristics that affect behavior and brain structure. We believe these factors should be further explored as points of intervention in prospective and/or randomized trials. For example, investing in proper centralized heating or other insulation facilities in homes may be beneficial for brain development and aging. Investigating the housing qualities identified by the World Health Organization as physical/mental health risks (wall thickness, shading from direct sunlight, natural and artificial ventilation)^65^ may also have neurobiological effects.

It is also worth noting that the use of multiple MRI facilities could also increase the generalizability of the results from this study. However, at the same time, we also recognize that this can also be a limitation. We addressed this by conducting multiple t-tests (Tables 2, 3) prior to the main analysis. The t-tests results showed significant mean differences in terms of FA-BHQ values. Therefore, the succeeding correlation and path analyses controlled for age, gender and experiment location. We believe this is still a reasonable procedure because whole brain measurements are not significantly altered. However, if specific brain regions are to be evaluated, more steps are needed in order to account for the difference in MRI facility^66,67^.

### Limitations and future work

The data used is cross-sectional in nature, therefore causal relationships and directionalities cannot be formed. Second, our sample only consists of Japanese individuals which could be prone to nationality bias. Changes in whole brain structures (gray matter volume and white matter integrity) may be different in other nationalities. Third, a larger number of samples may have increased the generalizability of the findings. Nevertheless, prospective and randomized investigations exploring the link between white matter integrity and actual behaviors using larger sample sizes and diverse nationalities are warranted in order to further elucidate the mechanisms connecting housing quality, sedentary behavior and anxiety. Fourth, the assessment of weekend/holiday sedentary behavior and anxiety were rather simplified. Future research utilizing more objective clinical evaluations of anxiety and sedentary are needed. Fifth, variables related to geographic location and income, which were not collected in this study, are also valid potential confounding variables. Including these variables in future experiments are also needed.

Finally, future RCTs and Longitudinal studies are also warranted in order to investigate the causal directionality and dose–response relationships among housing qualities, behavior, brain structure, and psychological outcomes. We believe this research direction may find its way to personalized healthcare and promote non-hospital/medicinal treatments of psychological and neurological disorders.

## Data Availability

All datasets generated for this study are available upon request.
